# Performance of an electronic health record-based phenotype algorithm to identify community associated methicillin-resistant *Staphylococcus aureus* cases and controls for genetic association studies

**DOI:** 10.1186/s12879-016-2020-2

**Published:** 2016-11-17

**Authors:** Kathryn L. Jackson, Michael Mbagwu, Jennifer A. Pacheco, Abigail S. Baldridge, Daniel J. Viox, James G. Linneman, Sanjay K. Shukla, Peggy L. Peissig, Kenneth M. Borthwick, David A. Carrell, Suzette J. Bielinski, Jacqueline C. Kirby, Joshua C. Denny, Frank D. Mentch, Lyam M. Vazquez, Laura J. Rasmussen-Torvik, Abel N. Kho

**Affiliations:** 1 Feinberg School of Medicine, Northwestern University, Chicago, IL USA; 2 Emory University School of Medicine, Atlanta, GA USA; 3 Biomedical Informatics Research Center, Marshfield Clinic Research Foundation, Marshfield, WI USA; 4 Marshfield Clinic Research Foundation, Marshfield, WI USA; 5 Geisinger Health System, Danville, PA USA; 6 Group Health Research Institute, Group Health Cooperative, Seattle, WA USA; 7 Mayo Clinic, Rochester, MN USA; 8 Department of Biomedical Informatics, Vanderbilt University, Nashville, TN USA; 9 The Center for Applied Genomics, Children’s Hospital of Philadelphia, Philadelphia, PA USA

**Keywords:** ca_MRSA, Phenotyping, Electronic Health Record, ca-MRSA Phenotype, GWAS

## Abstract

**Background:**

Community associated methicillin-resistant *Staphylococcus aureus* (CA-MRSA) is one of the most common causes of skin and soft tissue infections in the United States, and a variety of genetic host factors are suspected to be risk factors for recurrent infection. Based on the CDC definition, we have developed and validated an electronic health record (EHR) based CA-MRSA phenotype algorithm utilizing both structured and unstructured data.

**Methods:**

The algorithm was validated at three eMERGE consortium sites, and positive predictive value, negative predictive value and sensitivity, were calculated. The algorithm was then run and data collected across seven total sites. The resulting data was used in GWAS analysis.

**Results:**

Across seven sites, the CA-MRSA phenotype algorithm identified a total of 349 cases and 7761 controls among the genotyped European and African American biobank populations. PPV ranged from 68 to 100% for cases and 96 to 100% for controls; sensitivity ranged from 94 to 100% for cases and 75 to 100% for controls. Frequency of cases in the populations varied widely by site. There were no plausible GWAS-significant (*p* < 5 E −8) findings.

**Conclusions:**

Differences in EHR data representation and screening patterns across sites may have affected identification of cases and controls and accounted for varying frequencies across sites. Future work identifying these patterns is necessary.

**Electronic supplementary material:**

The online version of this article (doi:10.1186/s12879-016-2020-2) contains supplementary material, which is available to authorized users.

## Background

Methicillin-resistant *Staphylococcus aureus* is one of the most common causes of skin and soft tissue infections (SSTIs) in the United States [1]. Community-associated methicillin-resistant *Staphylococcus aureus* (CA-MRSA) has replaced traditional healthcare associated strains in many communities where it previously did not exist [2, 3]. Recent reports indicate that CA-MRSA strains contain more antibiotic resistance genes than previously encountered, and pose an enormous concern for patients, hospitals and public health entities [3]. Additionally, CA-MRSA strains express increased virulence factors leading to increased tissue destruction and more severe infections [4, 5]. A variety of genetic factors are suspected as a risk factor for recurrent CA-MRSA infection [4, 6, 7], with an increased prevalence in younger, healthier populations with no other identifiable risk factors [8].

The Centers for Disease Control and Prevention (CDC) definition of CA-MRSA distinctly differs from Healthcare-Associated MRSA (HA-MRSA). A soft-tissue infection is considered CA-MRSA if (1) a subsequently positive wound culture was taken within 48 h of hospital admission [9, 10], and (2) the patient did not have surgery, live in a long-term care facility, or undergo hemodialysis/peritoneal dialysis during the past year, and (3) the patient did not undergo catheterization or insertion of indwelling percutaneous devices during present hospital admission [3]. This definition was intended to clearly delineate community and hospital acquired (HA) infections, with the purpose of adequately differentiating the two phenotypes and potentially guiding empiric therapy [11]. However, it is not clear whether the criteria for CA-MRSA can readily translate into a computable phenotype using electronic health record (EHR) data (an increasingly common source for clinical data) which can then be used for genetic analysis.

Identifying disease phenotypes using EHR data has been a growing area of interest with the rapid increase in EHR adoption nationally [12, 13]. The Electronic Medical Records and Genomics (eMERGE) Network is a national consortium consisting of 9 funded sites (in phase II) formed to investigate the use of EHR systems for genetic research, in which phenotype identification algorithms can be proposed, disseminated and validated [14, 15]. The eMERGE Network has developed numerous phenotype algorithms using (EHR) data for use in genetic analyses [15–19]. In this paper, we describe the development and validation of a CA-MRSA case and control phenotype algorithm, implementation results and subsequent GWAS findings.

## Methods

### Algorithm development

**Fig. 1:**
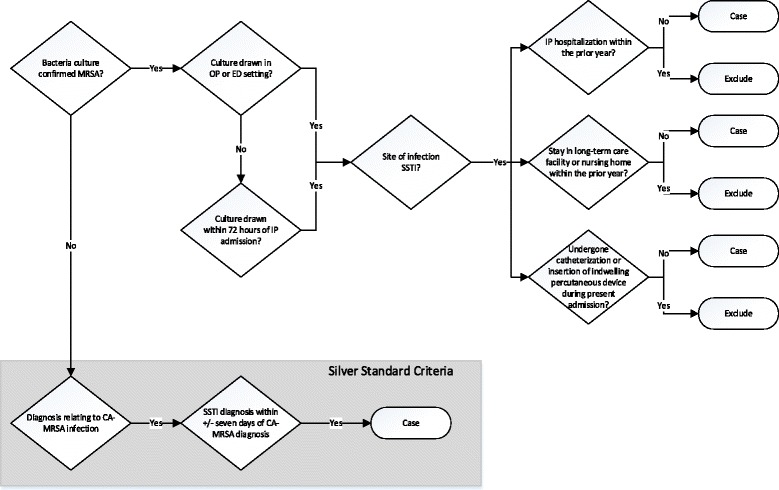
Algorithm for the identification of patients with CA-MRSA

**Fig. 2:**
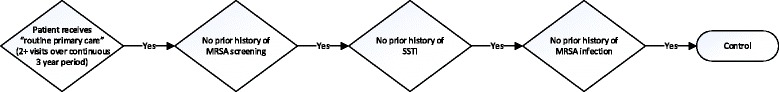
Algorithm for the identification of CA-MRSA controls

The CA-MRSA phenotype algorithm was based on the CDC definition and prior work in this space [3] and developed at Northwestern University (NU). Figures 1 and 2 show the case and control phenotype definitions, respectively. Case inclusion criteria included having a bacterial culture drawn from a skin and soft tissue (SSTI) infection site, in the outpatient or emergency department setting or within 72 h of admission to an inpatient setting, which confirmed a MRSA infection. It should be noted that the CDC definition considers MRSA to be HA if the infection occurs >48 h after admission, whereas we use ≥72 h in our definition to minimize the possibility of incorrectly categorizing CA as HA. Potential cases were excluded if the patient had a hospitalization in the prior year before the MRSA infection, a prior stay in a long term care facility or nursing home in the prior year, or had undergone catheterization or insertion of indwelling percutaneous devices during the admission in which MRSA was detected.

Given a recent clinical trend to empirically treat presumed SSTIs as CA-MRSA without drawing cultures [20], we also included “silver standard” criteria which removed the requirement for microbiology confirmed MRSA, and instead relied on clinical documentation of International Classification of Diseases, Ninth Revision (ICD-9) codes related to CA-MRSA infections, as well as the presence of a SSTI within a week of initial diagnosis, to determine case status (Fig. 1). All ICD-9 codes were based on the CDC definition for SSTIs associated with MRSA [21] (Additional file 1: Table S1).

Due to the complexity of the phenotype, multiple data inputs from clinical notes, records of past hospitalizations and laboratory culture results were needed to accurately define the phenotype. We leveraged prior work tracking MRSA within a health information exchange to create a list of the most common terms used in EHRs associated with SSTIs [22]. ICD-9 codes used to identify SSTIs were based on the CDC definition for SSTIs associated with MRSA [21].

Patients who had visited a primary care provider at least two times within a continuous 3-year period (i.e., received “routine primary care”) and had never had a positive MRSA screen, no prior history of an SSTI or any MRSA infection were considered controls (Fig. 2).

### Algorithm validation

The CA-MRSA phenotype algorithm performance was validated via manual chart review for a set number of randomly selected cases and controls, comparing the outcome of the phenotyping algorithm to the “gold standard” of individually abstracted information in the patient charts to verify the correctness of case or control status [18]. The algorithm was initially run, tested and validated on patients’ biobank data at NU. The final algorithm was then distributed to two other validating sites (Geisinger Health System and Marshfield Clinic) for implementation and validation. Implementation and selection of cases and controls for validation of the algorithm were completed using all patients’ data in each site’s biobank. Charts for 50 cases and 50 controls were reviewed at NU; 25 cases and 25 controls were reviewed at Geisinger; 25 cases and 25 controls were reviewed at Marshfield. Positive predictive value (PPV), negative predictive value (NPV) and sensitivity were calculated at each site individually. Additionally, at NU, all cases included via the silver standard criteria alone were validated through chart review. All statistical analyses were performed using SAS 9.4 (SAS Institute Inc., Cary, NC, USA).

### Phenotypic data

After outside site validation at Geisinger and Marshfield, the algorithm was distributed to the other eMERGE sites. In total, seven eMERGE institutions participated in this study (Northwestern University, Geisinger Health System, Marshfield Clinic, Children’s Hospital of Philadelphia (CHOP), Group Health Cooperative, Mayo Clinic and Vanderbilt University). Each site has robust genomic biobank projects linked with EHR data, stored in their site-specific data warehouses, for use in determining genotype-phenotype associations. For purposes of this study, patients with relevant EHR records were included only if all genetic data necessary for GWAS was also available. In addition to case and control status, age (at time of infection for cases and at time of last visit for controls), gender and race/ethnicity were also collected from the EHR.

### Genetic data

Details of the assembly of an imputed GWAS dataset for the eMERGE II Network have been published previously [23, 24]. In brief, SNPs were genotyped on a number of different platforms at different sites. Data were quality controlled at each site [25], then common SNPs were merged. SNPs were imputed to the 1000 Genomes Project phase 3 reference panel using IMPUTE [26]. Principal components fit to the pre-imputed SNP dataset were computed using EIGENSTRAT [27].

### Genetic analysis

GWAS analysis was run in SNPTest (version 2.4.1) [28] and included only those patients with phenotypes, eigenvectors and genetic information available. Models were stratified by race (African American (AA) and European American (EA) only, due to small sample sizes in all other groups) determined from principal components analyses. After completing analysis, we filtered on imputation information >0.8, minor allele frequency (MAF) ≥0.05, and Hardy-Weinberg Equilibrium (HWE) >0.000001 to remove spurious associations.

## Results

When implemented on the entire biobank population at each site, the CA-MRSA algorithm returned 124 cases and 1649 controls at NU, 76 cases and 2310 controls at Geisinger, and 61 cases and 7781 controls at Marshfield, without inclusion of the silver standard criteria. Table 1 summarizes the validation results from all three sites. PPV ranged from 68 to 100% for cases and 96 to 100% for controls; NPV ranged from 90 to 100% for cases and 80 to 100% for controls; sensitivity ranged from 94 to 100% for cases and 75 to 100% for controls. Application of the silver standard criteria did not yield significantly more patients at any of the validation sites. Only four additional cases at NU and four at Geisinger were identified after applying the silver standard criteria; no new patients were identified at Marshfield. Chart review of the “silver” cases at NU showed all four to be valid cases of CA-MRSA.

**Table 1:** Summary of chart review validation

	Cases			Controls		
	Northwestern	Geisinger	Marshfield	Northwestern	Geisinger	Marshfield
Total *N* ^a^	124	76	61	1649	2310	7781
Total Reviewed	50	25	25	50	25	25
Sensitivity	0.94	1	1	0.75	0.96	1
PPV	0.68	0.96	1	0.96	1	1
NPV	0.90	1	1	0.80	0.96	1

^a^Sample includes all patients in site’s biobank

Across all seven sites, the CA-MRSA phenotype algorithm (including the silver standard criteria) identified a total of 349 genotyped cases and 7761 genotyped controls among the biobank populations. Table 2 shows the breakdown of cases and controls by site. Addition of silver standard criteria yielded a small number of additional cases at some sites. Four sites (CHOP, Marshfield, Mayo Clinic and NU) added no “silver” *genotyped* cases (although, as described previously, some additional cases were discovered in the entire biobank population). The frequency of cases in the genotyped biobank sample also varied widely by site, ranging from 0.1% (CHOP) to 13.1% (Geisinger). Table 3 shows patient demographic characteristics, both overall and by case/control status for all adult eMERGE sites. The demographic breakdown of cases and controls by site and race can be found in Additional file 2: Table S2. The CHOP site contributed only 2 cases, and the demographics of cases and controls were very different from all other eMERGE sites; as such, GWAS analysis excluded data from this site. Among the resulting sites, 5111 patients (269 cases; 4842 controls) were European American and 770 (71 cases; 699 controls) were African American. Only 0.9% of cases and 0.8% of controls were identified as Hispanic or Latino in the EHR. The majority of cases and controls were female (52.4 and 61.9%) respectively. The average age for cases was 42 (SD = 22); the average age for controls was 67 (SD = 14).

**Table 2:** Summary of genotyped case and control subject counts^a^ by institution

	Overall	CHOP^b^	Geisinger	GHC	Marshfield	Mayo	NU	VU
Total Cases	349	2	34	39	15	1	62	196
Silver Only	40	0	1	1	0	0	0	38
Total Controls	7761	1869	233	1131	1871	306	783	1568
Prevalence	4.3%	0.1%	12.7%	3.3%	0.8%	0.3%	7.3%	11.1%

^a^Sample includes only GWAS genotyped patients from the site’s biobank

^b^CHOP data was not included in GWAS analysis

**Table 3:** Demographics of cases and controls in GWAS

	Overall	Cases	Controls
	*N* (%)	*N* (%)	*N* (%)
Total	6239	347 (5.6)	5892 (94.4)
Sex			
Male	2407 (38.6)	165 (47.6)	2242 (38.1)
Female	3832 (61.4)	182 (52.4)	3650 (61.9)
Ancestry			
European American	5111 (81.9)	269 (77.5)	4842 (82.2)
African American	771 (12.4)	72 (20.8)	699 (11.9)
Hispanic	14 (0.2)	0 (0)	14 (0.2)
Other	343 (5.5)	6 (1.7)	337 (5.7)
Ethnicity			
Hispanic or Latino	49 (0.8)	3 (0.9)	46 (0.8)
Not Hispanic or Latino	6076 (97.4)	340 (98.0)	5736 (97.4)
Unknown	114 (1.8)	4 (1.2)	110 (1.9)
Age (years) (mean(SD))	66 (16)	42 (22)	67 (14)

There were no plausible GWAS-significant (*p* < 5E-8) hits among the 269 cases and 4842 controls in European Americans or the 71 cases and 699 controls in African Americans (Additional file 3: Figure S1A and B). One apparent signal in African Americans on chromosome 6 included only very low frequency SNPs in a gene desert, suggesting that this result was a false positive. Examination of QQ plots and genomic inflation factors did not suggest any systematic inflation from the null distribution.

## Discussion

Development of an algorithm to capture a complex phenotype like CA-MRSA poses several challenges. First of all, the epidemiological definition of CA-MRSA by the CDC is complex and has several requirements that typically are not captured in structured data and instead must be extracted from clinical and laboratory notes. Therefore, our algorithm relied on each site’s ability to combine data from multiple EHR sources, including ICD-9 diagnosis codes, clinical notes, records of past hospitalizations and laboratory culture results in order to accurately extract phenotype cases and controls. The extraction of information from non-structured fields remains a significant obstacle to accurate phenotyping, suggesting the need for text-based strategies, such as national language processing, for phenotyping.

Second, differentiation between hospital and community-associated MRSA can be difficult. The issue is further complicated because of increasing presence of CA-MRSA strains in hospital settings and subcategories of HA-MRSA such as healthcare associated community onset (HACO) [29] and healthcare associated hospital onset (HAHO) [30] MRSAs. Our phenotype definition expands on previous epidemiologic studies by Casey et al. [31, 32], in which HA-MRSA is differentiated from CA-MRSA primarily by having an inpatient visit at the time of positive MRSA culture/diagnosis, an indwelling catheter or subcutaneous device at the time of positive MRSA culture/diagnosis, or a hospitalization, dialysis, surgery or residence in a nursing home within the year prior to a positive MRSA culture/diagnosis at a single healthcare system. Our study took place at seven institutions, spanning various EHR systems, each with unique complications to obtaining note-based requirements. Therefore, to increase the likelihood that the MRSA infection was community associated, we included a requirement that the site of infection must be an SSTI, in case of sparse clinical notes. Similarly, we required that any diagnosis of CA-MRSA be combined with an SSTI diagnosis within the week before or after the MRSA diagnosis. These differences may account for significant variation in performance of CA-MRSA case definitions, and as such, may also help explain the differences in frequency of cases across sites.

While our final cohort of patients included cases and controls from multiple institutions spanning urban, suburban and rural geographies across the country, each individual site’s cohort only included information from one institution. Prior work has demonstrated significant fragmentation of key data on MRSA across institutions [22, 33]. Indeed, during the validation process, chart reviewers noted that many patients not excluded from the case definition in the algorithm were found to have been hospitalized and/or underwent surgery at sites other than the institution where the CA-MRSA culture was drawn and, therefore, were incorrectly included as cases by the algorithm. Again, this supports the need for text-driven strategies in defining phenotypes, as well as effective health information exchange in regions in which overlap of patient populations across multiple institutions is significant.

The frequency of cases among the biobank populations varied widely between each site. This algorithm was run at a total of seven sites which collectively represent a diverse population of patients, clinicians, EHRs and conventions of documentation. While this variety enhances diversity of the population for analyses, it also provides a challenge to EHR extraction. Additionally, our study included only patients who also had genetic information captured as part of each institution’s biobank. This may account for some of the cross-site difference in case numbers used for analysis, particularly if biobanking efforts focused on specific populations. Our sites represent a sample of larger healthcare delivery institutions and are located in areas with differing rates of CA-MRSA [34]. Institutional differences in screening practices (e.g., mandated active surveillance of specific populations) for MRSA may have also accounted for cross site differences in the frequency of cases among the genotyped populations from the biobank [35, 36].

Selection of a set number of cases and controls for validation purposes (rather than selecting numbers proportional to the prevalence of each group in the study population) may have led to inflation of sensitivity estimates due to validation bias. Given the low frequency of MRSA in this population, we felt enrichment of cases for validation was necessary. As all sites validated the same ratio of cases and controls, we anticipate this inflation to be similar across all three validation sites.

Small sample size was a limitation of our study. Original application of the algorithm at the three validation sites (NU, Geisinger and Marshfield) returned only 112 valid cases to be used in the final GWAS. The ICD-9 based “silver” criteria were added in order to obtain additional cases to increase GWAS power. However, despite anecdotal evidence that front-line clinicians empirically treat all suspected CA-MRSA patients and do not routinely draw wound cultures, we identified very few new cases of CA-MRSA using ICD-9 codes alone that were not otherwise picked up by the original definition (*n* = 8 in the entire biobanked sample). Due to the small counts and 100% accuracy of patients obtained at NU, we did not require that other sites validate the “silver” algorithm. This criteria required an ICD-9 code for CA-MRSA, which clinicians may not often use to characterize SSTIs without the availability of confirmatory cultures (i.e., preference for the use of ICD-9 codes for “Abscess” or “Cellulitis” when culture has not been performed), and is largely physician dependent. The lack of frequent clinician documentation using these ICD-9 codes may be responsible for low patient numbers falling into this category and currently limits the utility of this approach. Further research into the EHR-based documentation patterns of clinicians for SSTIs that represent suspected CA-MRSA cases will help address these concerns.

The lack of GWAS-significant findings in either European or African Americans is disappointing, but not surprising given the limited sample size and anticipated modest effect size of any common genetic variants pre-disposing to CA-MRSA infection [6, 7, 37]. With CA-MRSA cases not defined in a uniform manner (as detailed above) and not screened for in a uniform manner (also detailed above), measurement error in the identification of cases would be expected to bias any association estimates between SNPs and CA-MRSA to the null. Despite these reports, differences in rates of host susceptibility to *Staphylococcus aureus* colonization and infection and differences in susceptibility to severity of related diseases point to a role for host genetic factors in susceptibility to CA-MRSA infections [6].

## Conclusion

The algorithmic extraction of CA-MRSA cases and controls from EHRs presents challenges and new possibilities for phenotypic-genotypic association studies. Our algorithm represents, to our knowledge, a first attempt at validating an otherwise complicated phenotype across multiple care sites. Variation in patient populations, screening practices, conventions of documentation and EHR data capture make standardization of an algorithm challenging and may account for variation in algorithm performance. Future work should focus on identifying these specific differences, as accounting for institutional variations when defining the algorithm may assist in identifying additional valid cases and controls to provide additional power to detect genetic risk factors predisposing carriers to CA-MRSA.

## Additional files

Additional file 1
**Table S1:** *Staphylococcus aureus* associated skin and soft tissue infections. (DOC 29 kb)
Additional file 2
**Table S2:** Demographics of genotyped cases and controls by site. (DOC 47 kb)
Additional file 3
**Figure S1:** A: CA-MRSA GWAS results in European Americans. B: CA-MRSA GWAS results in African Americans. (ZIP 294 kb)

## References

[1] Chua K (2011). Antimicrobial resistance: Not community-associated methicillin-resistant Staphylococcus aureus (CA-MRSA)! A clinician’s guide to community MRSA - its evolving antimicrobial resistance and implications for therapy. Clin Infect Dis.

[2] Elston DM (2009). How to handle a CA-MRSA outbreak. Dermatol Clin.

[3] Maree CL (2007). Community-associated methicillin-resistant Staphylococcus aureus isolates causing healthcare-associated infections. Emerg Infect Dis.

[4] Mediavilla JR (2012). Global epidemiology of community-associated methicillin resistant Staphylococcus aureus (CA-MRSA). Curr Opin Microbiol.

[5] Shukla SK (2005). Community-associated methicillin-resistant Staphylococcus aureus and its emerging virulence. Clin Med Res.

[6] Shukla SK, Rose W, Schrodi SJ (2015). Complex host genetic susceptibility to Staphylococcus aureus infections. Trends Microbiol.

[7] Ye Z (2014). Genome wide association study of SNP-, gene-, and pathway-based approaches to identify genes influencing susceptibility to Staphylococcus aureus infections. Front Genet.

[8] Sattler CA, Mason EO, Kaplan SL (2002). Prospective comparison of risk factors and demographic and clinical characteristics of community-acquired, methicillin-resistant versus methicillin-susceptible Staphylococcus aureus infection in children. Pediatr Infect Dis J.

[9] Benoit SR (2008). Community strains of methicillib-resistant *Staphlococcus aureus* as potential cause of healthcare-associated infections, Uruguay, 2002–3004. Emerg Infect Dis.

[10] Minnesota Department of Health. http://www.health.state.mn.us/divs/idepc/diseases/mrsa/camrsa/hcp.html. Accessed 30 June 2016.

[11] Millar BC (2007). Proposed definitions of community-associated meticillin-resistant Staphylococcus aureus (CA-MRSA). J Hosp Infect.

[12] Wei WQ (2012). Impact of data fragmentation across healthcare centers on the accuracy of a high-throughput clinical phenotyping algorithm for specifying subjects with type 2 diabetes mellitus. J Am Med Inform Assoc.

[13] Pathak J, Kho AN, Denny JC (2013). Electronic health records-driven phenotyping: challenges, recent advances, and perspectives. J Am Med Inform Assoc.

[14] McCarty CA (2001). The eMERGE Network: a consortium of biorepositories linked to electronic medical records data for conducting genomic studies. BMC Med Genomics.

[15] Gottesman O (2013). The Electronic Medical Records and Gemonimcs (eMERGE) network: past, present, and future. Genet Med.

[16] McCarty CA (2011). The eMERGE Network: a consortium of biorepositories linked to electronic medical records data for conducting genomic studies. BMC Med Genomics.

[17] Muthalagu A (2014). A rigorous algorithm to detect and clean inaccurate adult height records within EHR systems. Appl Clin Inform.

[18] Newton KM (2013). Validation of electronic medical record-based phenotyping algorithms: results and lessons learned from the eMERGE network. J Am Med Inform Assoc.

[19] Kho AN (2011). Electronic medical records for genetic research: results of the eMERGE consortium. Sci Transl Med.

[20] Mistry RD (2014). Clinical management of skin and soft tissue infections in the U.S. emergency departments. West J Emerg Med.

[21] McCaig LF (2006). Staphylococcus aureus-associated skin and soft tissue infections in ambulatory care. Emerg Infect Dis.

[22] Kho AN (2013). A regional informatics platform for coordinated antibiotic-resistant infection tracking, alerting, and prevention. Clin Infect Dis.

[23] Crosslin DR (2014). Controlling for population structure and genotyping platform bias in the eMERGE multi-institutional biobnak linked to electronic health records. Front Genet.

[24] Mosley JD (2015). A genome-wide association study identifieds variants in KCNIP4 associated with ACE inhibitor-induced cough. Pharmacogenomics J.

[25] Zuvich RL (2001). Pitfalls of merging GWAS data: lessons learned in the eMERGE network and quality control procedures to maintain hight data quality. Genet Epidemiol.

[26] Howie B (2012). Fast and accurate genotype inputation in genome-wide association studies through pre-phasing. Nat Genet.

[27] Patterson N (2006). Population structure and eigenanalysis. PLoS Genet.

[28] Wellcome Trust Case Control Consortium (2007). Genome-wide association study of 14,000 cases of seven common diseases and 3,000 shared controls. Nature.

[29] Lenz R (2012). The distinct category of healthcare associated bloodstreem infections. BMC Infect Dis.

[30] Wang SH (2015). Molecular and clinical characteristics of hospital and community onset methicillin-resitant Staphylococcus aureur strains associated with bloodstream infections. J Clin Microbiol.

[31] Casey JA (2013). A population-based study of the epidemiology and clinical features of methicillin-resistant Staphylococcus aureus infection in Pennsylvania, 2001–2010. Epidemiol Infect.

[32] Casey JA (2013). High-density livestock operations, crop field application of manure, and risk of community-associated methicillin-resistant Staphylococcus aureus infection in Pennsylvania. JAMA Intern Med.

[33] Kho AN (2008). Use of a regional health information exchange to detect crossover of patients with MRSA between urban hospitals. J Am Med Inform Assoc.

[34] Dukic VM, et al. Epidemics of community-associated methicillin-resistant Staphylococcus aureus in the United States: a meta-analysis. Otto M, ed. PLoS ONE. 2013;8(1):e52722. doi:10.1371/journal.pone.0052722. 10.1371/journal.pone.0052722 PMC3534721 23300988

[35] Lyles RD, et al. Regional epidemiology of methicillin-resistant Staphylococcus aureus among critically ill children in a state with mandated active surveillance. J Pediatric Infect Dis Soc. 2015. 10.1093/jpids/piv050 PMC8376206 26407280

[36] Kavanagh KT (2014). The use of surveillance and preventative measures for methicillin-resistant staphylococcus aureus infections in surgical patients. Antimicrob Resist Infect Control.

[37] Brown EL (2015). Genome-wide association study of Staphylococcus aureus carriage in a community-based sample of Mexican-Americans in Starr County, Texas. PLoS One.

