# Supplementary material for: Performance of an electronic health record-based phenotype algorithm to identify community associated methicillin-resistant Staphylococcus aureus cases and controls for genetic association studies
Source: BMC Infect Dis. 2016 Nov 17;16:684. doi: 10.1186/s12879-016-2020-2 (PMC5114817; doi:10.1186/s12879-016-2020-2)
Supplement: Additional file 1 — Table S1: Staphylococcus aureus associated skin and soft tissue infections. (DOC 29 kb) [file 12879_2016_2020_MOESM1_ESM.doc]

**Additional file 1: Table S1:** *Staphylococcus aureus* associated skin and soft tissue infections

| **Diagnosis** | **ICD-9-CM code** |
| --- | --- |
| Inflammatory disease of breast | 611 |
| Carbuncle and furuncle | 680.* |
| Cellulitis and abscess of finger and toe | 681.* |
| Other cellulitis and abscess | 682.* |
| Impetigo | 684.* |
| Unspecified local infection of skin and subcutaneous tissue | 686.9 |
| Other specified diseases of hair and hair follicles | 704.8 |
| Hydradenitis | 705.83 |

*** “wild card”**
