# Supplementary material for: Performance of an electronic health record-based phenotype algorithm to identify community associated methicillin-resistant Staphylococcus aureus cases and controls for genetic association studies
Source: BMC Infect Dis. 2016 Nov 17;16:684. doi: 10.1186/s12879-016-2020-2 (PMC5114817; doi:10.1186/s12879-016-2020-2)
Supplement: Additional file 2 — Table S2: Demographics of genotyped cases and controls by site. (DOC 47 kb) [file 12879_2016_2020_MOESM2_ESM.doc]

**Additional file 2: Table S2:** Demographics of genotyped^a^ cases and controls by site

|  | **N^a^** | **% Male** | **% EA** | **%AA** | **% Hispanic** | **Age Mean(SD)** |
| --- | --- | --- | --- | --- | --- | --- |
| **Cases** | 349 | 47.6 | 77.5 | 20.4 | 0.9 | 52.3 (18.7) |
| **CHOP^b^** | 2 | 25 | 75 | 25 | 0 | 7.8 (6.7) |
| **Geisinger** | 34 | 64.7 | 100 | 0 | 0 | 63.1 (14.4) |
| **GHC** | 39 | 53.9 | 87.2 | 2.6 | 5.1 | 73.9 (11.9) |
| **Marshfield** | 15 | 40 | 100 | 0 | 0 | 63.3 (11.6) |
| **Mayo** | 1 | 0 | 100 | 0 | 0 | 58.0 (NA) |
| **NU** | 62 | 32.3 | 75.8 | 24.2 | 0 | 54.2 (14.9) |
| **VU** | 196 | 49 | 70.9 | 28.6 | 0.5 | 44.7 (17.2) |
| **Controls** | 7,761 | 38.1 | 82.2 | 11.9 | 0.9 | 67.2 (14.1) |
| **CHOP^b^** | 1,869 | 55.9 | 59.4 | 34.3 | 2.0 | 11.4 (5.0) |
| **Geisinger** | 233 | 53.7 | 99.6 | 0 | 0.9 | 66.0 (15.5) |
| **GHC** | 1,131 | 46.2 | 90.6 | 4.8 | 1.7 | 77.7 (9.2) |
| **Marshfield** | 1,871 | 37.1 | 99.1 | 0 | 0.4 | 70.5 (10.4) |
| **Mayo** | 306 | 46 | 99 | 0.3 | 0.7 | 71.5 (11.6) |
| **NU** | 783 | 19.7 | 83.8 | 15.3 | 0 | 58.4 (13.2) |
| **VU** | 1,568 | 38.6 | 63.3 | 35 | 1 | 59.5 (14.8) |
| ^a^Sample includes only GWAS genotyped patients from the site's biobank | | | | | |  |
| ^b^CHOP data was not included in GWAS analysis | | | |  |  |  |
