# Supplementary figures and images for: Performance of an electronic health record-based phenotype algorithm to identify community associated methicillin-resistant Staphylococcus aureus cases and controls for genetic association studies
Source: BMC Infect Dis. 2016 Nov 17;16:684. doi: 10.1186/s12879-016-2020-2 (PMC5114817; doi:10.1186/s12879-016-2020-2)

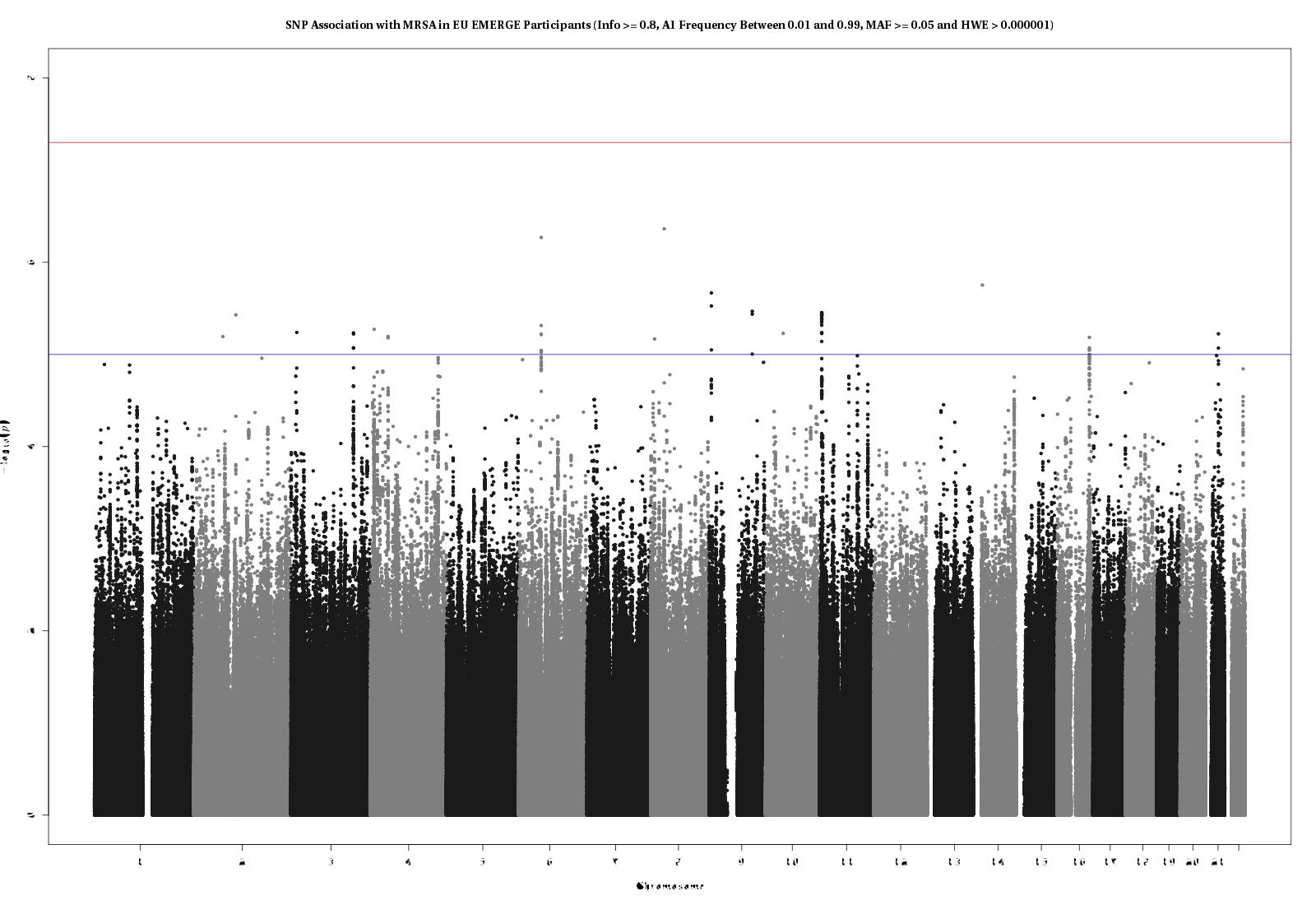

Supplement: Additional file 3 — Figure S1: A: CA-MRSA GWAS results in European Americans. B: CA-MRSA GWAS results in African Americans. (ZIP 294 kb) [file 12879_2016_2020_MOESM3_ESM.zip › Sup_Figure_1AR2.docx]

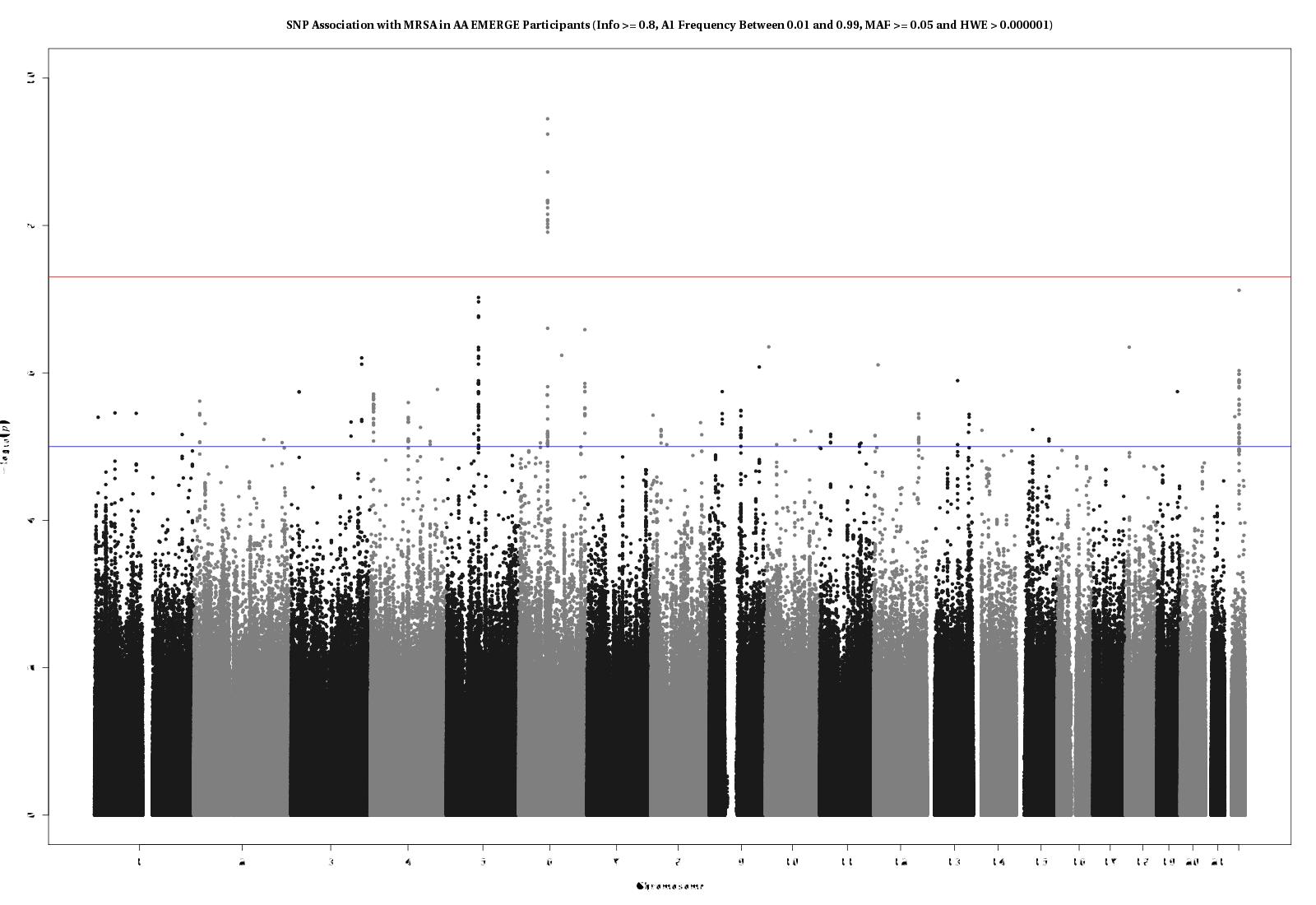

Supplement: Additional file 3 — Figure S1: A: CA-MRSA GWAS results in European Americans. B: CA-MRSA GWAS results in African Americans. (ZIP 294 kb) [file 12879_2016_2020_MOESM3_ESM.zip › Sup_Figure_1BR2.docx]
